# Clinical and Multimodal Imaging Features of Choroidal Nevi in the Korean Population

**DOI:** 10.3390/jcm11226666

**Published:** 2022-11-10

**Authors:** Chul Hee Lee, Hansang Lee, Seung Min Lee, Eun Young Choi, Junwon Lee, Min Kim

**Affiliations:** Department of Ophthalmology, Gangnam Severance Hospital, Yonsei University College of Medicine, Seoul 06273, Korea

**Keywords:** choroidal nevus, multimodal imaging, choroidal malignant melanoma

## Abstract

Choroidal nevus is a precursor of choroidal melanoma. Multimodal imaging has become vital in predicting the malignant transformation of choroidal nevi. This single-center, retrospective study analyzed clinical characteristics and multimodal imaging findings of 168 choroidal nevi (164 patients) of the Korean population. The mean age at presentation was 50 ± 15 (range, 13–85) (women, *n* = 88 [53.7%]). Choroidal nevi (melanotic, *n* = 164 [97.6%]; postequatorial, *n* = 160 [95.2%]) were densely located between the optic disc and foveola (65.5%). The mean maximum linear basal diameter on fundus photography and thickness on optical coherence tomography were 2.97 ± 1.51 mm and 521 ± 297 μm, respectively. On ultrasonography, the mean thickness was 0.87 ± 0.60 mm. Choroidal nevi in women were associated with a higher maximum linear basal diameter (3.23 ± 1.65 vs. 2.68 ± 1.21 mm; *p* = 0.033) and age at diagnosis (52 ± 14 vs. 47 ± 16 years; *p* = 0.046) than those in men. Choroidal nevi with associated subretinal fluid (23.2%) presented with larger basal diameter (*p* = 0.049) and thickness on B-scan and optical coherence tomography (*p* < 0.001), but a younger age at diagnosis (*p* < 0.001) than those of dry nevi. This multimodal imaging study of choroidal nevi revealed some distinct characteristics, including topographic distribution, sex-related differences, and a younger age at diagnosis of nevi with fluid.

## 1. Introduction

A choroidal nevus is defined as an unequivocal, pigmented (slate blue or green–gray; melanotic) or nonpigmented (amelanotic) choroidal lesion with a diameter of at least 500 μm [1]. Histopathologically, a benign choroidal nevus typically shows proliferation of pigmented spindle cells in the choroid, with low mitotic activity [2]. Although most choroidal nevi have a benign clinical course, the annual rate of malignant transformation of a choroidal nevus has been estimated to be 1 in 8845 among the white population in the USA [3]. Most large-scale studies on choroidal nevi have been based on the white population. Racial differences should be considered when performing studies on choroidal nevi because a substantial difference in the prevalence of choroidal nevi and choroidal melanoma among different ethnic groups has been reported in previous studies [4,5,6,7]. In a cross-sectional study of a multiethnic cohort, the prevalence of choroidal nevi were 4.1%, 1.2%, 0.7%, and 0.4% among whites, Hispanics, blacks, and Chinese, respectively [4]. In other Asian population-based studies, the prevalence of choroidal nevi ranged from 1.4% to 2.9% [8,9].

According to previous studies, although the prevalence of choroidal nevi was lower among Asians, the features of choroidal nevi on fundus photographs were similar to those in Caucasians [8,10]. However, in the non-Caucasian group (African–American, Hispanic, Asian, Asian Indian, Middle Eastern, and unknown), some distinctions, including a lower age at presentation, a higher prevalence of dysplastic nevus syndromes, and a reduced association with previous cutaneous melanoma, were present [10]. Moreover, non-Caucasians with choroidal nevus had more symptoms and fewer nevi per eye. However, since the non-Caucasian group included various ethnicities and only 15 East Asians were included in this study, it could not accurately represent the East Asian population. In the Singapore Malay Eye Study (SiMES), there was no definite difference in clinical features of choroidal nevi on fundus photography between Asian and Caucasian populations [8]. However, the Asians included in this study were a group of mixed ethnicities of Southeast Asians and East Asians. In another study based on an entirely Chinese population, You et al. investigated fundus photography characteristics of choroidal nevi in 75 subjects over a 5-year follow-up period and reached similar conclusions that choroidal nevi of Asians had benign characteristics with little growth [11]. Although these studies have revealed some characteristics of choroidal nevi in the Asian population, a further study with a longer follow-up period and detailed multimodal imaging is required to understand the typical clinical characteristics of choroidal nevi in Asians.

Further, although many epidemiologic studies have been performed on choroidal nevi in the Asian population, no published data regarding multimodal imaging characteristics of choroidal nevi in Asians exist. After major risk factors for malignant transformation of choroidal nevi were identified using multimodal imaging [12], multimodal imaging has become crucial in early detection of patients at risk. Therefore, we aimed to investigate the clinical and multimodal imaging features of choroidal nevi in patients who were referred to two tertiary health care centers in South Korea.

## 2. Materials and Methods

### 2.1. Study Participants

This retrospective, cross-sectional study adhered to the tenets of the Declaration of Helsinki and was approved by the institutional review board of Gangnam Severance Hospital. Considering the retrospective design, the requirement of informed consent was waived. We used the U-Severance electronic medical records program to screen patients diagnosed with a choroidal nevus at the Gangnam Severance Hospital and Shinchon Severance Hospital from January 2006 to October 2021. Multimodal imaging of all potential patients with choroidal nevi was primarily reviewed by two retinal specialists (C.L. and H.L.). After reviewing the images, the specialists excluded patients who did not meet the diagnostic criteria of choroidal nevus according to the following definition as per the Blue Mountains Eye Study (BMES): an unequivocal, pigmented (slate blue or green–gray) choroidal lesion of at least 500 µm in diameter [1]. Choroidal lesions resembling nevi that were partly or largely depigmented were defined as amelanotic nevi and were included in the study. The following pigmented lesions were excluded: optic disc nevi (melanocytomas), congenital hypertrophy of retinal pigment epithelium, and pigmented scars. The final diagnosis of choroidal nevus was confirmed by two senior experienced retinal specialists (J.L. and M.K.) in consensus.

Demographic and clinical data, including age, race, sex, presenting symptoms, and best-corrected visual acuity (BCVA) of all patients were collected. We reviewed their medical histories, blood work-up results, and laboratory test results to check for a previous history of cancer, dysplastic nevus syndrome, and cutaneous melanoma.

### 2.2. Clinical Evaluation

All included eyes were evaluated using color fundus photography with the ultra-widefield Optos fundus camera (Optos PLC, Dunfermline, UK) and KOWA VX-20 retinal fundus camera (Kowa Company Ltd., Tokyo, Japan). We evaluated the lesion’s color (melanotic or amelanotic), maximum linear basal diameter (MLBD), location and distance relative to the optic disc and fovea. For the distance between the nevus and the optic disc, we measured from the center of the optic disc to the center of the nevus. The center of the nevus was defined by the point where vertical and horizontal lengths intersect. Furthermore, for each choroidal nevus, we manually plotted a scatter plot of the topographical distribution of the center of the choroidal nevi relative to the center of the optic disc. Having used two different fundus camera systems, we calibrated the quantitative measurements by assuming that the horizontal diameter of the optic disc was 1.77 mm [13]. No cases of an unusually small or large disc that could affect the calibration were present. Moreover, to avoid the Mercator projection issue when quantifying the lesion size and distance of peripheral choroidal nevus, we utilized the ProViewTM (Optos PLC, Dunfermline, UK) software to accurately reduce peripheral distortion [14]. We also analyzed and recorded the presence of associated features, including halo, drusen, and changes in retinal pigment epithelium (RPE).

### 2.3. Multimodal Imaging

#### 2.3.1. Optical Coherence Tomography

Spectral-domain optical coherence tomography (OCT) was performed using Spectralis OCT (Heidelberg Engineering GmbH; Heidelberg, Germany). In most patients, enhanced depth imaging (EDI) mode was used to capture the posterior margin of the choroidal nevus. All images were captured, centered, and focused at the deepest part of the choroidal nevus, thus enabling adequate visualization of structures posterior to RPE and intralesional anatomy. For most choroidal nevi, we used a 5.7 × 4.3 mm volume scan, with 37 B-scan images. For larger choroidal nevi, the volume scan was adjusted to fit all of the choroidal nevi. To evaluate the characteristics of the whole nevus, the raster scan was used over the entire nevus. The presence of subretinal fluid (SRF) or intraretinal fluid (IRF) was checked. The regularity and thickness of RPE over the choroidal nevi were compared to the RPE surrounding the choroidal nevus lesion. The thicknesses of the choriocapillaris, the choroid layer at the locations occupied by the choroidal nevus, and the choroidal nevus itself on OCT were measured by using the caliper tool. Since choroidal nevus is an intrachoroidal lesion, we measured from the posterior margin of the RPE (Bruch’s membrane) to the posterior margin of the choroidal nevus itself as the measurement of choroidal thickness. For analysis of the thickness of choroidal nevi, only choroidal nevi with visible posterior border were included.

#### 2.3.2. Fundus Autofluorescence Imaging

Fundus autofluorescence (AF) images were acquired using blue or green excitation light on flash-based fundus camera or confocal scanning laser ophthalmoscope systems (Spectralis HRA; Heidelberg Engineering GmbH; Heidelberg, Germany). AF images were taken at a 30° scan angle centered on the choroidal nevi. Images were graded for the presence of hypoautofluorescent, hyperautofluorescent, or stippled autofluorescent features of the lesion. Further, the presence of orange pigment (a risk factor for malignant transformation) was checked.

#### 2.3.3. Fundus Near-Infrared Imaging

We acquired fundus near-infrared reflectance (NIR) images (Spectralis HRA; Heidelberg Engineering GmbH; Heidelberg, Germany) and evaluated the reflectance of the choroidal nevus as either isoreflective, bright, or dark. NIR images were taken at a 30° angle centered at the choroidal nevi.

#### 2.3.4. Ultrasonography

We performed ultrasonography (Eye Cubed; Ellex; Adelaide, Australia) using a 10 MHz B-scan and analyzed the echogenicity and thickness of the lesions. A thickness above 2.0 mm was noted as a risk factor for malignant transformation.

#### 2.3.5. Fluorescein Angiography and Indocyanine Green Angiography

Fluorescein angiography (FA) and indocyanine green angiography (ICGA) were performed (Spectralis HRA; Heidelberg Engineering GmbH; Heidelberg, Germany) at a 30° scan angle centered at the choroidal nevi. Although there were no definite indications for performing FA or ICGA, these procedures were performed in patients with one or more risk factors for the malignant transformation of choroidal nevi (thickness above 2.0 mm on B-scan, symptomatic patients, associated fluids on OCT, an orange pigment on AF, a hollowness on ultrasonography, and a diameter above 5.0 mm on fundus photography). We recorded the presence of early and late hyperfluorescence and hypofluorescence. Moreover, the presence of choroidal neovascularization and other abnormal vessels were checked in cases of choroidal nevi with SRF or IRF.

### 2.4. Statistical Analysis

The mean, median, and standard deviation values of the evaluated variables were reported using descriptive statistics. Having performed Shapiro–Wilk test and plotted histogram, we checked that all parameters followed the Gaussian distribution. Thus, we performed parametric tests for the statistical analysis. We conducted an unpaired t-test and paired *t*-test for unpaired parameters and longitudinal analysis, respectively. Qualitative and quantitative measurements were performed by two masked graders (S.L. and E.C.). The intraclass correlation coefficient (ICC) was calculated for quantitative measurements to verify the agreement between the masked graders. Cohen’s kappa coefficient was calculated for inter-rater agreement for qualitative assessments. If the two masked graders disagreed on the qualitative assessment, adjudication was conducted by senior experienced retinal specialists (J.L. and M.K.) to settle the argument. In case of bilateral involvement of choroidal nevi, we used both eyes’ nevi data when performing statistics about individual nevi (MLBD on fundus photography, thicknesses on ultrasonography, OCT, AF image, NIR image, and FA/ICGA). When performing statistics related to demographics (sex, age, past medical history, and total follow-up period), we excluded the eye with smaller choroidal nevi to avoid duplication of data. Statistical analysis was performed by C.L. and M.K. Statistical analysis software SPSS ver. 21.0 (SPSS Inc., Chicago, IL, USA) was used to perform all statistics. All quantitative data are presented as mean ± standard deviation unless indicated otherwise.

## 3. Results

### 3.1. Patient Disposition and Demographic Data

A total of 168 choroidal nevi of 164 patients were included in the study. All patients were ethnically homogenous (Korean, 100.0%). Four patients had bilateral involvement. The mean ± standard deviation value of the age at diagnosis was 50 ± 15 years. The median age was 51 years (range: 13–85 years). There were 76 (46.3%) men and 88 (53.7%) women. No statistically significant difference was noted in sexual predilection. The mean ± standard deviation value of BCVA at presentation was 0.08 ± 0.14 logMAR (20/24 by Snellen equivalent). The mean ± standard deviation value of the spherical equivalent was −1.12 ± 2.55 D (range: −11.00 D to +2.75 D). The mean follow-up period was 2.8 ± 3.3 years (range: <1.0–15.8 years; median: 1.0 year). Ninety-one (55.5%) patients did not have any systemic disease. A previous history of cancer was present in seven (4.3%) patients (two breast, two lung, two thyroid, and one bladder cancer). None of the patients had a history of dysplastic nevus syndrome and cutaneous melanoma (Table 1).

### 3.2. Clinical Findings on Fundus Examination

**Figure 1:**
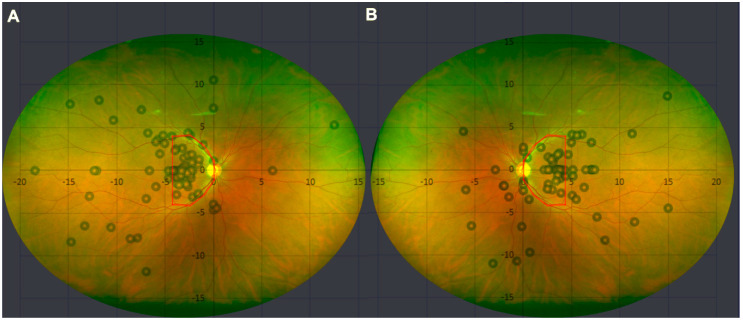
Topographical distribution of choroidal nevi in the Korean population. (**A**) Central points of 92 choroidal nevi of the right eye are plotted on an x–y graph with the optic nerve at the center (0,0). The positive and negative values on the x-axis are nasal and temporal to the optic nerve, respectively. The positive and negative values on the y-axis are superior and inferior to the optic nerve, respectively. The numbers represent the values of length in mm. Out of 92 choroidal nevi, 60 choroidal nevi were located within 4.7 mm temporal, superior, or inferior to the optic nerve (65.2%, red area). Eighty-seven choroidal nevi were located postequatorially (94.6%). (**B**) Central points of 76 choroidal nevi of the left eye are plotted on an x–y graph with the optic nerve at the center (0,0). The positive and negative values on the x-axis are temporal and nasal to the optic nerve, respectively. The positive and negative values on the y-axis are superior and inferior to the optic nerve, respectively. The numbers represent the values of length in mm. Out of 76 choroidal nevi, 50 choroidal nevi were located within 4.7 mm temporal, superior, or inferior to the optic nerve (65.8%, red area). Seventy-four choroidal nevi were located postequatorially (97.4%).

Of the 168 lesions, 164 (97.6%) lesions were melanotic, and four (2.4%) were amelanotic. Drusen was present in 60 (35.7%) lesions. A halo was present in 21 (12.5%) lesions. Regarding the location of choroidal nevi relative to the optic nerve, the choroidal nevi were located mostly inferotemporal (*n* = 53, 31.5%) and superotemporal (*n* = 45, 26.8%), followed by temporal (*n* = 42, 25.0%), inferonasal (*n* = 11, 6.5%), superior (*n* = 6, 3.6%), inferior (*n* = 4, 2.4%), nasal (*n* = 3, 1.8%), and superonasal (*n* = 2, 1.2%), to the optic nerve. The topographical distribution map of the choroidal nevi based on the distance from the optic nerve showed temporal predominance (Figure 1). Further, choroidal nevi were mostly found between the optic nerve and fovea (*n* = 110, 65.5%). Nearly all lesions were postequatorial (*n* = 160, 95.2%). The mean ± standard deviation value of the distance from the choroidal nevus to the optic nerve and foveola was 4.85 ± 3.95 mm and 4.10 ± 3.78 mm, respectively, and that of MLBD was 2.97 ± 1.51 mm (Table 2).

### 3.3. Optical Coherence Tomography Findings of Choroidal Nevi

OCT findings were available for 125 lesions of 122 patients. Most lesions had no fluid compartments (*n* = 88, 70.4%). SRF was present in 26 (20.8%) cases, and IRF was present in eight (6.4%) cases. Three (2.4%) lesions had both SRF and IRF overlying the choroidal nevi.

Segmentation analysis revealed that choroidal nevi caused certain changes in RPE. The RPE above the choroidal nevi was irregular and disrupted in majority of cases (*n* = 85, 68.0%). RPE thickness was increased (*n* = 65, 52.0%) or normal (*n* = 54, 43.2%). Choriocapillaris crowding and thinning was noted in most cases compared to adjacent choriocapillaris (relative thinning: *n* = 121, 96.8%), with an average thickness of 95 ± 52 μm. The posterior margin of the choroidal nevus was visible in 105 lesions. The maximum choroidal thickness of the area occupied by the choroidal nevus was mostly greater than the adjacent choroid (*n* = 100, 95.2%), with an average thickness of 617 ± 308 μm. The average choroidal nevus thickness on OCT was 521 ± 297 μm (range: 82 μm–1837 μm) (Table 3).

### 3.4. Other Multimodal Imaging Findings of Choroidal Nevi

AF images of 107 lesions were available. The AF imaging results of choroidal nevi were heterogeneous. Forty-eight (44.9%) lesions displayed isoAF, while 28 (26.2%) lesions showed hypoAF, and four (3.7%) lesions displayed hyperAF. Twenty-seven (25.2%) lesions displayed mixed hyperAF and hypoAF. Six choroidal nevi had orange pigment (5.6%).

NIR images of 128 lesions were available. Most of the choroidal nevi had bright features on NIR images (*n* = 109, 85.2%). There were seven (5.4%) isoreflective, six (4.7%) dark, and six (4.7%) mixed bright and dark lesions.

On B-scan ultrasonography (*n* = 115), most of the choroidal nevi revealed solid echogenicity (*n* = 112, 97.4%). The average thickness of the lesion was 0.89 ± 0.60 mm (range: 0.30 mm–2.50 mm).

FA images were available for 82 patients. The results of early FA were heterogenous, with 38 (46.3%) lesions displaying hypofluorescence, 20 (24.4%) lesions displaying hyperfluorescence, 17 (20.7%) lesions displaying no definite changes, and seven (8.5%) lesions displaying mixed hypo and hyperfluorescence. On late FA, 42 (51.2%) lesions displayed hyperfluorescence, 30 (36.6%) lesions displayed hypofluorescence, five (6.1%) lesions displayed no definite changes, and five (6.1%) lesions displayed mixed hypo and hyperfluorescence. Choroidal neovascularization was found in two cases (2.4%).

**Figure 2:**
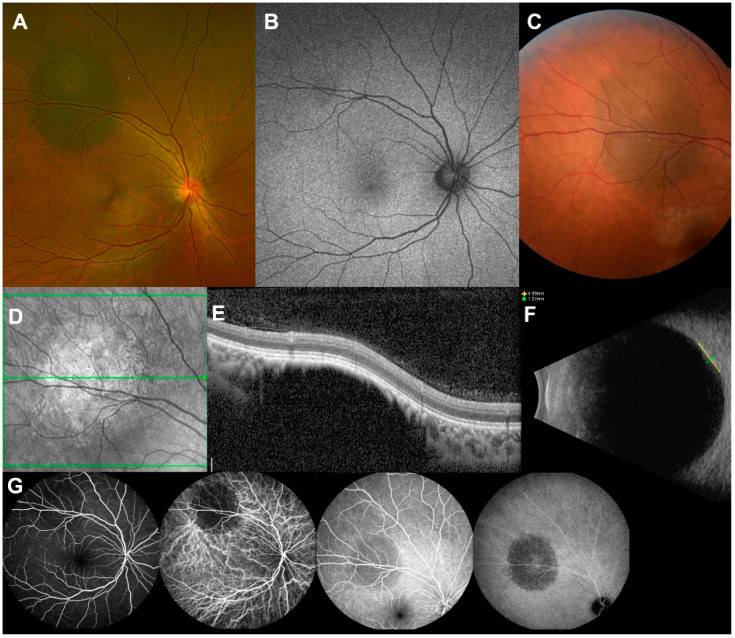
Multimodal imaging of a benign choroidal nevus of a Korean patient. A 21-year-old female patient was referred for an incidental finding of a choroidal tumor in the right eye. (**A**) Wide fundus photography shows a dark pigmented relatively flat 6.0 × 5.2 mm sized intraocular mass, 4.7 mm away from the optic disc and 3.4 mm away from the foveola. (**B**) Autofluorescence image shows a relatively isoautofluorescent mass at superior arcade. (**C**) Color fundus photography shows a greyish charcoal colored mass. (**D**) Near-infrared image shows a bright reflectance at the center of the mass. (**E**) Optical coherence tomography shows a hyporeflective intrachoroidal mass with 1105 μm thickness. Choriocapillaris over the mass seems compressed and thinned showing hyper-reflectance. (**F**) B-scan ultrasonography shows an echodense choroidal mass with 1.27 mm thickness. (**G**) Fluorescence angiography and indocyanine green angiography show a hypofluorescence at the early and late phase. With the diagnosis of a benign choroidal nevus with one risk factor (diameter over 5.0 mm), she was regularly followed-up under surveillance for malignant change.

ICGA images were available for 74 patients. All choroidal nevi displayed hypofluorescence on early ICGA (*n* = 74, 100.0%). On late ICGA, 65 (87.8%) lesions displayed hypofluorescence, and nine (12.2%) lesions displayed mixed hypo- and hyperfluorescence. Table 4 details these multimodal imaging findings. Figure 2 shows a representative multimodal imaging of benign choroidal nevi of a Korean patient.

### 3.5. Multimodal Imaging Findings of Choroidal Nevi Based on Sex

**Figure 3:**
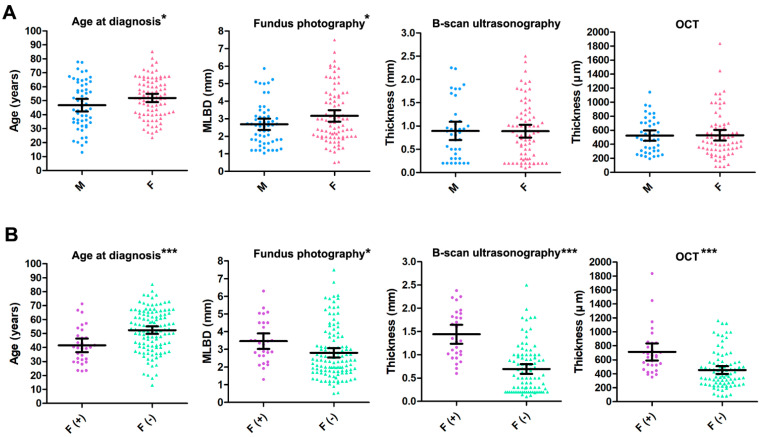
Clinical characteristics and multimodal imaging findings of choroidal nevi in the Korean population based on sex and the presence of subretinal fluid. (**A**) An unpaired t-test for characteristics of choroidal nevi in the Korean population based on sex. The mean age at diagnosis was significantly higher in women with choroidal nevi than in men with choroidal nevi (52 ± 14 years vs. 47 ± 16 years; *p* = 0.046). The mean maximum linear basal diameter (MLBD) of the choroidal nevi on fundus photography was significantly higher in women with choroidal nevi than that in men with choroidal nevi (3.23 ± 1.65 mm vs. 2.68 ± 1.21 mm; *p* = 0.033). However, the mean thickness of choroidal nevi on B-scan ultrasonography (0.89 ± 0.60 mm vs. 0.89 ± 0.61 mm; *p* = 0.969) or optical coherence tomography (OCT) (529 ± 327 μm vs. 524 ± 241 μm; *p* = 0.935) were not significantly different between women and men with choroidal nevi. (**B**) An unpaired *t*-test for characteristics of choroidal nevi in the Korean population based on the presence of subretinal fluid. The mean age at diagnosis was significantly lower in patients with fluid-positive choroidal nevi than in patients with dry choroidal nevi (42 ± 13 years vs. 52 ± 15 years; *p* < 0.001). On fundus photography, the mean MLBD of fluid-positive choroidal nevi was significantly higher than that of dry choroidal nevi (3.45 ± 1.17 mm vs. 2.85 ± 1.56 mm; *p* = 0.049). The mean thickness of fluid-positive choroidal nevi was significantly higher than that of dry choroidal nevi on B-scan ultrasonography (1.44 ± 0.52 mm vs. 0.70 ± 0.51 mm; *p* < 0.001) and OCT (712 ± 325 μm vs. 454 ± 258 μm; *p* < 0.001). F, female; F (+), choroidal nevi with subretinal fluid; F (−), choroidal nevi without fluid compartments; M, male; MLBD, maximum linear basal diameter; OCT, optical coherence tomography. Data are presented as mean ± standard deviation. The center line indicates the mean and error bars indicate 95% confidence intervals. * *p* < 0.05, *** *p* < 0.001.

An unpaired t-test for characteristics of choroidal nevi in the Korean population based on sex showed that the mean age at diagnosis was significantly higher in women than in men (52 ± 14 years vs. 47 ± 16 years; *p* = 0.046). The mean MLBD of the choroidal nevi on fundus photography was significantly higher in women than in men (3.23 ± 1.65 mm vs. 2.68 ± 1.21 mm; *p* = 0.033). However, the values of the mean thickness of choroidal nevi on B-scan ultrasonography (0.89 ± 0.60 mm vs. 0.89 ± 0.61 mm; *p* = 0.969) and OCT (529 ± 327 μm vs. 524 ± 241 μm; *p* = 0.935) were not significantly different between women and men (Figure 3A).

### 3.6. Multimodal Imaging Findings of Choroidal Nevi Based on the Presence of Subretinal Fluid

An unpaired *t*-test for characteristics of choroidal nevi in the Korean population based on the presence of subretinal fluid showed that the mean age at diagnosis was significantly lower in patients with fluid-positive (F [+]) choroidal nevi than in those with fluid-negative (F [−]) choroidal nevi (42 ± 13 years vs. 52 ± 15 years; *p* < 0.001). On fundus photography, the mean MLBD was significantly higher for F (+) choroidal nevi than for F (−) choroidal nevi (3.45 ± 1.17 mm vs. 2.85 ± 1.56 mm; *p* = 0.049). The values of the mean thickness on B-scan ultrasonography (1.44 ± 0.52 mm vs. 0.70 ± 0.51 mm; *p* < 0.001) and OCT (712 ± 325 μm vs. 454 ± 258 μm; *p* < 0.001) were significantly higher for F (+) choroidal nevi than for F (−) choroidal nevi (Figure 3B). There was no significant difference in sex regarding subretinal fluid (X^2^ = 0.28 *p* = 0.675; Chi-square test).

## 4. Discussion

To the best of our knowledge, this is the first study describing the clinical and multimodal imaging characteristics of choroidal nevi in an entirely East Asian population, specifically of Korean ethnicity. Topographically, choroidal nevi in this population showed temporal dominance with respect to the optic nerve (Figure 1). Moreover, nearly all lesions were postequatorial (*n* = 160, 95.2%). Finding choroidal nevi more commonly at the postequatorial area than in the periphery may be due to the practicality of screening the posterior pole. Many quiet lesions may have been missed due to difficulty of screening peripheral choroidal lesions. However, previous studies based on large scale autopsy of healthy eyes have shown a predilection of choroidal nevi being found in the posterior pole [15,16]. There may be a certain predilection of the choroidal nevus formation in the posterior pole. In a previous study of the Caucasian population, a similar temporal dominance with respect to the optic nerve with the density of lesion centers gradually decreasing with distance from the macula was observed [17]. In this previous study, the distribution of choroidal nevi in the temporal and nasal areas to the foveal center was almost even. However, in the present study based on the Korean population, the center of the choroidal nevi were heavily concentrated in the nasal to foveal center between the optic cup center and foveola (*n* = 110, 65.5%). The reason behind the distribution of choroidal nevi of the temporal and nasal side of the fovea seems unclear. However, a recent study of healthy Japanese subjects using polarization-sensitive OCT showed significantly higher concentration of choroidal melanin-containing tissue at the temporal to foveal center compared to that of the nasal side [18]. Choroidal melanin is known to absorb stray light and to have protective role against uveal melanoma [19,20,21]. The high density of choroidal melanin pigments at the temporal to fovea center may be more protective to the formation of choroidal nevus and choroidal melanoma at the temporal to foveal center, therefore leading to higher concentration of choroidal nevi at the nasal to foveal center of our East Asian cohort. Further studies need to be performed to identify whether similar topographical distribution of choroidal melanin pigment is observed in the Korean population. Additionally, white-population-based studies of choroidal melanin pigment distribution need to be reviewed to identify if there are any racial differences in topographic distribution of the choroidal melanin pigment. Furthermore, there is a possible location bias since the area between the optic disc and foveola would be readily seen on a fundus exam and would be captured in a standard macular OCT. Further studies with a larger sample size may be needed for detailed validation.

The OCT findings of choroidal nevi of the Korean population shows some similarities to those of Caucasian-based studies. Compared to a large-scale study of a Caucasian population conducted by Shields et al. [22], there were similar features regarding the preservation of inner retinal layers, and the gradual disruption of outer retinal and RPE layers. However, the mean thickness of Korean choroidal nevi was thinner [521 μm (range: 82–1837)] than that of the Caucasian-based population [685 μm (range: 184–1643)]. Choriocapillaris compression was noted in both studies.

Although the prevalence of choroidal nevi in male and female were statistically insignificant, some distinctions in clinical findings and multimodal imaging results between men and women with choroidal nevi existed in our data. Similar to previous studies, the sex ratio of choroidal nevi was almost equal in our database [1,4,5,8,11,23]. However, some subtle distinctions in clinical findings and multimodal imaging results between men and women with choroidal nevi existed (Figure 3A). First, the mean MLBD at initial presentation was slightly larger in women with choroidal nevi (3.23 ± 1.65 mm vs. 2.68 ± 1.21 mm; *p* = 0.033). Another notable distinction was slightly higher mean age at presentation in women with choroidal nevi than that of men with choroidal nevi (52 ± 14 years vs. 47 ± 16 years; *p* = 0.046). This may imply that sex hormones may influence the growth and development of choroidal nevi. In 2015, Qui M et al. demonstrated a significant relationship between increased total lifetime of unopposed estrogen and the incidence of choroidal nevi and melanoma [24]. Other studies have evaluated adjunctive targeted therapy with agents such as tamoxifen for the treatment of uveal melanoma [25]. A larger initial MLBD of choroidal nevi and older age at diagnosis in female patients may show that prolonged exposure to unopposed estrogen may be an important factor influencing the growth and formation of choroidal nevi in women. However, the sex-related differences in thicknesses of choroidal nevi measured on OCT and ultrasonography and in the development of SRF and IRF were insignificant. We speculate that female sex hormones may contribute to increased MLBD and age at diagnosis of choroidal nevi in women; however, further studies are required to elucidate the relationship between female sex hormones and these factors.

The multimodal imaging results showed that choroidal nevi with fluid compartments were larger and thicker than those without fluid compartments (Figure 3B). The MLBD of choroidal nevi with fluid was significantly larger than that of dry choroidal nevi (*p* = 0.049). Choroidal nevi with fluid had a higher thickness than that of those without fluid on B-scan ultrasonography (*p* < 0.001) and OCT (*p* < 0.001). Although the thickness on OCT may be influenced less by subretinal or intraretinal fluid, the thickness on B-scan ultrasonography may be overestimated by the presence of fluid compartments [22]. Therefore, measurement of nevus thickness by OCT may be preferred in cases with fluid compartments for exact measurement. It was also interesting to note that the age at diagnosis of patients with F (+) choroidal nevi was lower than that of patients with dry choroidal nevi (*p* < 0.001). Although a previous study of predominantly white population showed that choroidal nevi with fluid was more prevalent in older population [26], our findings may be a notable differential characteristic of choroidal nevi of the East Asian race. The age at diagnosis of choroidal melanoma has been reported to be significantly younger in the East Asian population than that of Caucasian race [27]. The mean age at diagnosis for uveal melanoma decreases from 59–62 years of age in Caucasians, to 55 years in Japanese, 51 years in Taiwanese, and 45 years in Chinese populations [28,29,30,31,32]. These results suggest that genetic factors may be stronger risk factors for the development of uveal melanoma in East Asian races rather than environmental factors. Additionally, since F (+) choroidal nevi may induce visual symptoms and signs such as decreased visual acuity, visual field defects, and floaters, patients may visit the eye clinic earlier, and thus, the diagnosis is confirmed at a younger age than that of those with dry choroidal nevi. The younger age of diagnosis of F (+) choroidal nevi may be a distinct characteristic of Korean choroidal nevi and implicates that other ethnicity-related factors rather than environmental factors may presumably play an important role in determining the mitotic activity and malignant potential of a choroidal nevus [26]. Therefore, careful observation and follow-up may be required when choroidal nevi are found at an early age.

The limitations of this study were the retrospective nature and the selection bias of tertiary referral hospitals, where cases of more severe, complicated, and larger choroidal nevus than that present in the general population might have been selected for analysis. Many mild, small, and flat types of choroidal nevi might not have been included in this study since local clinics did not refer cases of small choroidal nevi regularly to the study hospitals. Additionally, since we have included choroidal nevi sized above 500 μm, some very small choroidal nevi may have been missed [4]. Due to the retrospective nature of the study, multimodal imaging results could not be obtained for all patients. Due to invasiveness of the procedure, FA and ICGA were performed for patients with features prone to malignant transformation. Therefore, the results of FA and ICGA of this cohort may have selection bias. However, we believe that for this disease entity, which is not fully clarified in the East Asian population, the numbers of patients and lesions included are sufficient to obtain valid statistical results. Among the multimodal imaging, OCT angiography could not be obtained for most of the patients since it was a novel imaging modality and was not performed routinely for choroidal nevi. However, previous studies of OCT angiography in choroidal nevi have shown that OCT angiography may be a powerful imaging tool that may tell the difference between a malignant melanoma and a choroidal nevus by analyzing choroidal vascular flow rate [33,34,35]. OCT angiography of different types of choroidal nevi would provide deeper insight about its pathophysiology and malignant change potentials. Using artificial intelligence (AI) and machine learning to diagnose choroidal nevi and their malignant transformation risks solely based on fundus photographs may be challenging [36]. However, by integrating the database of multimodal imaging of choroidal nevi, there may be substantial potential in applying AI and machine learning in diagnosis and malignant transformation prediction of choroidal nevi [37].

## 5. Conclusions

In conclusion, this is the first Korean-population-based study of choroidal nevi and multimodal imaging features. Novel findings of the study were the following. First, the topographical distribution of choroidal nevi showed that the lesions were densely located between the optic nerve and foveola. Second, choroidal nevi in women had a higher MLBD and were diagnosed at an older age at presentation than those in affected men. Third, F (+) choroidal nevi showed a higher MLBD and thickness on ultrasonography and OCT and a lower age of the patients at diagnosis than dry choroidal nevi. The present study demonstrated some distinct and interesting characteristics of choroidal nevi in the Korean population. Further studies on malignant transformation rates of choroidal nevi in the Korean cohort are required to evaluate the value of multimodal imaging in the management of choroidal nevi in East Asians.

## Figures and Tables

**Table 1:** Choroidal Nevi (*n* = 168) in 164 Patients: Demographic Findings.

Variable	Value
Age at diagnosis (years), mean ± SD (median [range])	50 ± 15 (51 [13–85])
Sex, *n* (%) Male Female	76 (46.3%) 88 (53.7%)
Best-corrected visual acuity at presentation, mean ± SD	0.08 ± 0.14 logMAR 20/24 Snellen
Spherical equivalent (D), mean ± SD (median [range])	−1.12 ± 2.55 (−0.50 [+2.75–−11.00])
Systemic disease, *n* (%) None Hypertension Diabetes mellitus Cancer Others	91 (55.5%) 36 (22.0%) 23 (14.0%) 7 (4.3%) (2 Breast, 2 Lung, 2 Thyroid, 1 Bladder) 31 (18.9%)
Total follow-up period (years), mean ± SD (median [range])	2.8 ± 3.3 (1.0 [<1.0–15.8])

D, diopter; SD, standard deviation. Data are presented as number (%) unless otherwise indicated.

**Table 2:** Choroidal Nevi (*n* = 168) in 164 Patients: Initial Clinical Findings on Fundus Examination.

Variable	Value
Color, *n* (%) Melanotic Amelanotic	164 (97.6) 4 (2.4)
Drusen, *n* (%)	60 (35.7)
Halo, *n* (%)	21 (12.5)
Location relative to the optic nerve, *n* (%) Inferior Superior Nasal Temporal Inferonasal Inferotemporal Superonasal Superotemporal	4 (2.4) 6 (3.6) 3 (1.8) 42 (25.0) 11 (6.5) 53 (31.5) 2 (1.2) 45 (26.8)
Postequatorial location, *n* (%) Beyond the equator, *n* (%)	160 (95.2) 8 (4.8)
Distance to the optic nerve (mm), mean ± SD	4.85 ± 3.95
Distance to the fovea (mm), mean ± SD	4.10 ± 3.78
Maximum linear basal diameter (mm), mean ± SD	2.97 ± 1.51

SD, standard deviation. Data are presented as number (%) unless otherwise indicated.

**Table 3:** Choroidal Nevi (*n* = 125) in 122 Patients: Optical Coherence Tomography Findings.

Variable	Value			
**Fluid Compartment, *n* (%)** **None** **Subretinal fluid** **Intraretinal fluid** **Mixed subretinal and intraretinal fluid**	88 (70.4) 26 (20.8) 8 (6.4) 3 (2.4)			
**Neurosensory layers status, *n* (%)** **Internal limiting membrane** **Nerve fiber layer** **Ganglion cell layer** **Inner plexiform layer** **Inner nuclear layer** **Outer plexiform layer** **Outer nuclear layer** **External limiting membrane** **Ellipsoid layer**	Normal 124 (99.2) 123 (98.4) 122 (97.6) 120 (96.0) 119 (95.2) 98 (78.4) 79 (63.2) 67 (53.6) 53 (42.4)	Absent 0 (0) 0 (0) 0 (0) 0 (0) 0 (0) 0 (0) 0 (0) 0 (0) 0 (0)	Thinning 1 (0.8) 1 (0.8) 2 (1.6) 4 (3.2) 5 (4.0) 23 (18.4) 42 (33.6) 56 (44.8) 48 (38.4)	Thickening 0 (0) 1 (0.8) 1 (0.8) 1 (0.8) 1 (0.8) 4 (3.2) 4 (3.2) 2 (1.6) 24 (19.2)
**Retinal pigment epithelium, *n* (%)** **Regularity** **Thickness relative to adjacent retinal pigment** **epithelium**	Regular 40 (32.0) Normal 54 (43.2)	Irregular 85 (68.0) Absent 0 (0)	Thinning 6 (4.8)	Thickening 65 (52.0)
**Choriocapillaris over nevus** **Thickness relative to adjacent choriocapillaris, *n* (%)** **Thickness (μm), mean ± SD**	Normal 3 (2.4) 95 ± 52	Absent 0 (0)	Thinning 121 (96.8)	Thickening 1 (0.8)
**Choroid (*n* = 105)** **Thickness relative to adjacent choroid, *n* (%)** **Thickness (μm), mean ± SD**	Normal 5 (4.8) 617 ± 308	Absent 0 (0)	Thinning 0 (0)	Thickening 100 (95.2)
**Choroidal nevus thickness (*n* = 105) (μm), mean ± SD**	521 ± 297 (range: 82–1837)			

OCT, optical coherence tomography; SD, standard deviation. Data are presented as number (%) unless otherwise indicated.

**Table 4:** Fundus Autofluorescence Imaging, Near-Infrared Reflectance Imaging, B-Scan Ultrasonography, Fluorescein Angiography, and Indocyanine Green Angiography Findings of Choroidal Nevi.

Variable	Value			
**Fundus AF *n* (%) (*n* = 107)** **AF** **Orange pigment (+)**	IsoAF 48 (44.9) 6 (5.6%)	HyperAF 4 (3.7)	HypoAF 28 (26.2)	Mixed 27 (25.2)
**NIR, *n* (%) (*n* = 128)**	Isoreflective 7 (5.4)	Bright 109 (85.2)	Dark 6 (4.7)	Mixed 6 (4.7)
**B-scan ultrasonography (*n* = 115)** **Echogenicity, *n* (%)** **Thickness (mm), mean ± SD**	Solid 112 (97.4) 0.87 ± 0.60	Hollow 3 (2.6) (range: 0.30–2.50)		
**FA, *n* (%) (*n* = 82)** **Early** **Late**	Normal 17 (20.7) 5 (6.1)	HyperF 20 (24.4) 42 (51.2)	HypoF 38 (46.3) 30 (36.6)	Mixed 7 (8.5) 5 (6.1)
**ICGA, *n* (%) (*n* = 74)** **Early** **Late**	Normal 0 (0) 0 (0)	HyperF 0 (0) 0 (0)	HypoF 74 (100) 65 (87.8)	Mixed 0 (0) 9 (12.2)

AF, autofluorescence; FA, fluorescein angiography; HyperF, hyperfluorescence; HypoF, hypofluorescence; ICGA, indocyanine green angiography; NIR, near-infrared reflectance; SD, standard deviation. Data are presented as number (%) unless otherwise indicated.

## Data Availability

The data presented in this study are available on request from the corresponding author. The data are not publicly available due to privacy issues.
